# Examining prosocial tendencies in men who have sex with men: Potential impacts on blood donation following the 2023 reform

**DOI:** 10.1111/vox.70256

**Published:** 2026-03-31

**Authors:** Antonia Leiße, Michel Clement

**Affiliations:** ^1^ Social Research Impact Lab, Institute for Marketing University of Hamburg Hamburg Germany

**Keywords:** MSM, propensity score matching, prosocial behaviour

## Abstract

**Background and Objectives:**

Men who have sex with men (MSM) historically faced legal restrictions in accessing blood donation systems. While recent policy changes aim to reduce discriminatory barriers, empirical evidence on prosocial engagement patterns among MSM remains limited. This study examines whether MSM differ from heterosexual men in their prosocial engagement in monetary donations, in‐kind support, caretaking and volunteering, and whether these differences vary across age.

**Materials and Methods:**

We rely on data from the Socio‐Economic Panel (SOEP), which is a representative survey of the German population. MSM are compared to heterosexual men with respect to prosocial behaviour using propensity score matching. We estimate regression models, including interaction terms between sexual orientation and age, to assess the differences in prosocial engagement.

**Results:**

MSM exhibit a higher likelihood of engaging in money donations and in‐kind support compared to heterosexual men. When accounting for interaction effects of sexual orientation and age, we find differences in in‐kind support, caretaking and the amount of monetary donations. These differences decrease with increasing age. Although a higher proportion of MSM is engaged in in‐kind support, there are no differences in the amount of money donated for in‐kind support. We find no differences between the groups with respect to volunteering.

**Conclusion:**

MSM represent a relevant target group for non‐profit engagement, particularly in the domains of financial (amount) and in‐kind contributions (likelihood). Blood establishments should leverage recent reforms to build trust through inclusive communication and diversity training. Alternative engagement channels can enhance social participation for those deferred for medical reasons.

**Highlights**

- Men who have sex with men (MSM) and heterosexual men differ in their background characteristics, underscoring the importance of applying a matching approach.
- MSM show a higher likelihood of engaging in monetary and in‐kind donations.
- Interaction effects show higher MSM engagement in money donations (amount), in‐kind support and caregiving, with the differences diminishing with age.

## INTRODUCTION

One blood donation holds the potential to save up to three lives [1]. However, blood is considered a scarce resource. In 2024, several countries, including the United States and Germany, experienced severe blood shortages, leading to postponed medical procedures and heightened public concern [2, 3]. This scarcity is compounded by the fact that blood donations can come only from human donors who meet stringent safety criteria to protect the recipients from any potential risks. Historically, these medical guidelines have led to the exclusion of certain groups, such as men who have sex with men (MSM), due to their classification as a higher risk population group. This policy, while intended to safeguard recipients, has been widely debated for its impact on the available donor pool and its alignment with evolving understandings of risk and equality [4].

Emergency situations have led to legislative changes aimed at addressing supply challenges, leading to the re‐admission of previously excluded population groups. Allowing MSM to donate blood represents an opportunity to increase the donor pool.

In recent years, some countries have revised their blood donation policies to focus less on sexual orientation and more on individual behavioural risk factors. This shift aims to expand the donor pool while maintaining safety. In early 2023, Germany enacted a reform of the Transfusion Act (Transfusionsgesetz), ending the categorical deferral of MSM. Prior to this, German guidelines imposed a mandatory 4‐month deferral on all MSM following sexual contact with a new or multiple male partners, regardless of the specific sexual practice.

According to the updated guidelines, deferral criteria are now based solely on individual risk: donors are deferred for 4 months only if they have changed sexual partners frequently (two partners or more) or engaged in specific high‐risk practices, such as anal intercourse with a new partner, regardless of the gender of the partner [5].

The question arises as to whether previous blood donation regulations have led to the exclusion of particularly ‘valuable donors’, who may exhibit higher levels of prosocial behaviour as suggested by some behavioural theories [6]. Behavioural differences between homosexual and heterosexual individuals have been examined, for example, in marketing research. Notably, a meta‐analysis conducted by Eisend and Hermann [7] have identified differences in consumption patterns among younger individuals, which tend to diminish with age. However, it remains unclear whether these differences extend to prosocial behaviours.

We aim to compare the prosocial behaviour of MSM and heterosexual men by using the data from the German Socio‐Economic Panel (SOEP). SOEP is a representative panel that includes more than 20,000 individuals annually. Exploiting the first inclusion of sexual orientation in 2019 and detailed measures of prosocial engagement in 2020, we compare monetary donations, in‐kind support, volunteering and caregiving activities. These behaviours serve as proxies for potential blood donation engagement, as participation in one form of prosocial activity is associated with higher involvement in other prosocial domains [8, 9].

Our findings show that a higher proportion of MSM is engaged in monetary donations and in‐kind support after matching. When accounting for interaction effects of age and sexual orientation and applying inverse probability weights, the differences in in‐kind support engagement and in the amount of monetary donations decrease.

### Conceptualization and related literature

Consumer behaviour research has extensively examined homosexual consumers, documenting differences in behaviours and preferences compared to heterosexual individuals [7, 10]. However, their engagement in prosocial behaviour remains less understood. Prosocial behaviour differs from consumption in that it involves actions intended to benefit others or society [11]. Understanding prosocial tendencies is particularly relevant for blood donation, which is strongly motivated by altruistic motives [12].

Our research builds on Wilson's [6] kin‐selection hypothesis, which posits that homosexual behaviour may have evolved as an adaptive strategy to enhance inclusive fitness. Based on the theory, homosexual individuals may compensate for a lack of direct reproductive gain by contributing to the well‐being of their kin or broader community, highlighting altruistic tendencies [6, 13]. Research indicates that homosexual men report stronger altruistic values than heterosexual men [14]. Homosexual men are also more likely to engage in caregiving roles than heterosexual men [15]. Moreover, a study conducted by Perry et al. [16] used neuroimaging to evaluate gender and sexual orientation differences in empathy‐related brain activations. Their results show that heterosexual women exhibit the highest empathy‐related activation, followed by homosexual men. The authors conclude that the attraction to men rather than sexual orientation itself is associated with greater empathy [16].

Research on MSM and blood donation has progressed in two main directions. First, studies examine MSM's attitudes towards blood donation, including reasons for and against donating [17, 18]. Thus, MSM display a strong willingness to donate blood despite historical and regulatory prohibitions [17, 19]. Despite donation bans, a substantial proportion of MSM report engaging in blood donation, with reported rates ranging from 17% to 38.7% [14, 20].

Second, research focuses on the consequences of allowing MSM to donate blood, including simulations that model the residual risk of human immunodeficiency virus (HIV)‐contaminated blood units when the deferral period would be reduced from 12 months to 4 [21] and 3 months [22]. Moreover, Tiberghien et al. [23] provided epidemiological evidence showing the effect of lifting MSM deferral periods in France. While MSM donors showed higher relative risks for certain infectious markers compared to other donor groups, these estimates should be interpreted cautiously because of the small size of the MSM donor population [23].

Little is known about whether MSM exhibit higher prosocial engagement, which may influence donation intentions independently of eligibility rules. Additionally, research on MSM behaviour is often based on samples that are not representative of the broader MSM population. Rather, Coffman et al. [24] have shown that conventional surveys that rely on self‐reported statements underestimate the size of the lesbian, gay, bisexual, and transgender population, their attitudes and behaviour. We address these gaps using representative German data and propensity score (PS) matching to ensure comparability between MSM and heterosexual men.

This study therefore addresses the following question:

RQ 1 Do MSM and heterosexual men differ in their engagement in prosocial behaviours, such as monetary donations, in‐kind support, volunteering and caregiving?

According to Cass [25], homosexual identity formation is characterized by six stages: Confusion, Comparison, Tolerance, Acceptance, Pride and Synthesis. Younger MSM are more likely than older MSM to be at an earlier developmental stage, which is characterized by some degree of identity uncertainty, high levels of self‐monitoring and strong desires for either uniqueness (distinctiveness) or group identification (community signalling). It is during these developmental stages that MSM engage in behaviours that will typically vary from those exhibited by heterosexual males as they explore and address stigma, seek a sense of belonging and/or develop and begin to express their developing identities. When MSM move through developmental stages towards later stages—specifically identity acceptance and identity synthesis—their sexual orientation becomes part of a more integrated and stable self‐concept. With continued identity integration, MSM's sexual orientation continues to play a decreasingly important role in influencing their daily behaviour, thereby resulting in a reduction of the need to signal or differentiate their identity. Therefore, MSM's behavioural and attitudinal differences that are typically more pronounced during adolescence and young adulthood tend to become less pronounced as they continue to age. This has also been highlighted by Eisend and Hermann [7] in a marketing context. They find that homosexual consumers generally exhibit slightly higher consumption‐favouring behaviours, such as increased spending, positive brand attitudes and openness to innovation, compared to heterosexual consumers. With increasing age, MSM's consumption preferences become more similar to those of heterosexual men [7]. Building on this reasoning, the present study addresses a second research question:

RQ 2 Do potential differences in prosocial engagement between MSM and heterosexual men persist across age, or do they diminish over the life course?

## MATERIALS AND METHODS

### Dataset

In this study, we compare prosocial behaviour between MSM and heterosexual men across four domains: (i) monetary donations, (ii) in‐kind support, (iii) volunteering and (iv) caregiving. We rely on the SOEP, a representative longitudinal survey of private households in Germany.

The dataset, launched in 1984, is the longest running longitudinal survey of private households in Germany and employs a multi‐stage, regionally clustered random sampling design and collects data via annual interviews (paper‐and‐pencil personal interview/computer‐assisted personal interview) [26]. To maintain high retention and representativeness, participants receive a financial compensation [27]. It is well suited for examining complex social, economic and behavioural questions because of its extensive thematic scope and long‐running data collection. Each year, the SOEP gathers responses from up to 30,000 participants on a wide range of topics.

In 2019, sexual orientation was surveyed for the first time. We use the variables sexual orientation and sex for the classification of MSM based on the former blood donation eligibility criteria (act of transfusion) in Germany. We classified bisexual and homosexual individuals whose sex is male as MSM. We wish to note that one's sexual orientation does not necessarily mean that an individual is sexually active. The present comparison is explicitly restricted to cisgender men to isolate differences that can be attributed to sexual orientation rather than gender identity.

Our dataset consists of men whose sex is male and who (i) are either homosexual or bisexual (*n* = 388) and (ii) heterosexual (*n* = 9454). The 2020 dataset encompasses various prosocial activities, including (i) money donations, (ii) in‐kind donations (providing goods, gifts or clothing), (iii) caretaking and (iv) volunteering. We analyse these behaviours in the context of 2020, when MSM were not allowed to donate blood without restrictions in Germany.

### Statistical analyses

To compare the difference of sexual orientation on our outcome variables, we need to control for selection effects by applying PS matching. This approach is particularly advantageous in observational studies, as it facilitates a more balanced comparison between non‐randomized groups by mimicking the properties of a randomized experiment [28]. The PS is estimated via logistic regression and is defined as the probability of being in the treatment group (MSM) given a set of covariates that affect both group belonging (MSM or not) and the outcomes of interest (prosocial behaviour). Thus, the PS reflects how likely it is that an individual belongs to the treatment group based on a given set of covariates, including socio‐demographic, psychographic and health‐related factors.

By applying the common support assumption, observations in the treatment group that fall outside the minimum and maximum range of PS observed in the control group are excluded. As a result, individuals with similar PS are matched to each other, thereby reducing pre‐existing differences and improving comparability [29].

We test two matching algorithms, namely nearest neighbour matching with replacement and Epanechnikov kernel with a bandwidth of 0.6. Nearest neighbour matching pairs each treated unit with the control unit whose PS is closest. When matching is performed with replacement, the same control unit may serve as the nearest neighbour for multiple treated units [30].

For kernel matching, control units contribute to estimating the counterfactual for the treated unit only when they are within 0.6 units in terms of the propensity score distance (PSD) of the treated unit and will have a smooth decline in weights as PSD increases from that point. All observations above the bandwidth receive a weight of zero. The bandwidth provides an optimal balance between achieving high efficiency and comparable estimates [31]. The application of these matching algorithms resulted in comparable results, supporting the robustness of our findings (Tables A3, A4, A5, A6).

To estimate the PS, a set of covariates was included in the logistic regression model. The selection of these variables was informed by existing literature on differences between MSM and heterosexual men. The operationalization of all variables is provided in Table A1, and the literature‐based inclusion of covariates is provided in Appendix 2. The matched datasets were used to conduct *t*‐tests to examine differences in the relevant prosocial domains. In addition, logistic regression models including an interaction term between sexual orientation and age were used to examine whether the engagement varies with age. The same interaction structure was implemented in two linear regression models, in which the log‐transformed amounts of in‐kind support and monetary donations served as dependent variables. We use the logarithm of the donation amount because the donation amounts are highly right‐skewed. This makes the results more stable and yields measures that better reflect proportional differences in donation behaviour [32]. All analyses were weighted using PS‐based weights to improve balance between MSM and heterosexual men.

## RESULTS

### Sample description

Table 1 provides an overview of the distribution of characteristics among heterosexual and MSM pre‐matching.

**TABLE 1:** Sample descriptives and group specific comparisons.

		Heterosexual men		MSM			
		*M*/*n*	SD/%	*M*/*n*	SD/%	*t*‐value/chi^2^	*p*
Socio‐demographic	Age	51.93	17.14	45.81	16.50	6.90	<0.001
	Married (yes)	5946	63.0%	124	32.1%	149.66	<0.001
	New federal state (yes)	2308	24.4%	129	33.3%	15.61	<0.001
	Children (yes)	3042	32.2%	40	10.3%	82.86	<0.001
	Labour earnings last year	44,340	137,103	33,262	47,503	1.59	0.112
	Full‐time employment (yes)	5206	55.1%	184	47.4%	8.79	0.003
	Unemployment (yes)	433	4.6%	22	5.7%	1.00	0.317
Psychographic factors	Political interest	2.60	0.85	2.90	0.87	−6.79	<0.001
	Risk affinity	5.52	2.23	5.49	2.18	0.23	0.822
	Positivity	5.73	1.17	5.44	1.43	4.64	<0.001
	Reciprocity	6.38	0.90	6.50	0.78	−2.75	0.006
	Costly reciprocity	5.49	1.31	5.60	1.29	−1.62	0.105
	Perceived social determination	4.36	1.45	4.55	1.46	−2.55	0.011
	General worry index	1.83	0.33	1.82	0.31	0.48	0.630
	Satisfaction with health	6.83	2.09	6.90	2.19	−0.71	0.479
	Current satisfaction	7.58	1.59	7.50	1.58	0.98	0.327
	Mood index	2.25	0.64	2.38	0.68	−4.00	<0.001
Health‐related factors	Chronic illness (yes)	3694	39.2%	173	44.6%	4.51	0.034
	Smoking (yes)	2331	24.7%	121	31.2%	8.40	0.004
	Hours of sleep weekdays (h/day)	6.92	1.16	6.91	1.26	0.12	0.906
	Body mass index	27.26	4.72	26.64	5.48	2.51	0.012

MSM were substantially younger than heterosexual men (*M* = 45.81 vs. 51.93, *p* < 0.001). They were less likely to be married (32.1% vs. 63.0%, *p* < 0.001) and less likely to have children (10.3% vs. 32.2%, *p* < 0.001).

In terms of psychosocial characteristics, MSM show higher levels of political interest (*p* < 0.001) and report a more positive attitude towards themselves (*p* < 0.001). Moreover, MSM report significantly higher perceptions that life opportunities are determined by social circumstances (*M* = 4.55 vs. 4.36, *p* = 0.011).

Regarding physical and health‐related factors, a higher proportion of MSM reports having a chronic illness (44.6% vs. 39.2%, *p* = 0.034) and being smokers (31.2% vs. 24.7%, *p* = 0.004). In addition, MSM exhibit a significantly lower body mass index (BMI) compared to heterosexual men (*M* = 26.64 vs. 27.26, *p* = 0.012).

Overall, the sample descriptives highlight pre‐existing differences between MSM and heterosexual men and the importance of accounting for potential selection effects. Therefore, a logistic regression model with sexual orientation as the dependent variable is employed for each dependent variable to derive the corresponding PS (Table A2).

### RQ 1: Engagement rates in prosocial behaviour

Table 2 presents differences in prosocial engagement between MSM and heterosexual men before and after Epanechnikov kernel matching. Before matching, a higher proportion of MSM is found to donate money compared to heterosexual men (Mean_treated_ = 52%; Mean_control_ = 45%). This difference persists after matching (Mean_treated_ = 52%; Mean_control_ = 43%), indicating a robust and persistent gap in money donation behaviour even after adjusting for selection effects.

**TABLE 2:** Group comparison results for engagement in prosocial behaviour before and after matching (Epanechnikov kernel algorithm).

		MSM (treated)	Heterosexual men (control)	Diff.	SE	*t*
Money donation (yes/no)	Mean_before_	0.52	0.45	0.06	0.03	2.50**
	Mean_after_	0.52	0.43	0.09	0.03	3.37***
	*N*	381	9138			
In‐kind support (yes/no)	Mean_before_	0.31	0.21	0.11	0.02	5.10***
	Mean_after_	0.31	0.22	0.09	0.02	3.81***
	*N*	381	9115			
Caretaking (yes/no)	Mean_before_	0.07	0.06	0.01	0.01	0.88
	Mean_after_	0.07	0.06	0.02	0.01	1.11
	*N*	381	9159			
Volunteering (yes/no)	Mean_before_	0.16	0.17	−0.01	0.02	−0.34
	Mean_after_	0.16	0.17	0.00	0.02	−0.04
	*N*	377	9117			
Log amount of money donated (in €)	Mean_before_	5.12	5.23	−0.11	0.11	−1.00
	Mean_after_	5.12	4.87	0.25	0.11	2.17*
	*N*	198	4106			
Log amount of in‐kind donated (in €)	Mean_before_	5.84	6.08	−0.24	0.12	−2.08*
	Mean_after_	5.84	5.82	0.02	0.12	0.14
	*N*	119	1775			

Abbreviation: MSM, men who have sex with men.

* *p* < 0.05;

** *p* < 0.01;

*** *p <* 0.001.

We find a similar pattern for in‐kind support. Prior to matching, a higher proportion of MSM is seen to provide in‐kind support than heterosexual men (Mean_treated_ = 31%; Mean_control_ = 21%). This difference slightly decreases after matching (Mean_treated_ = 31%; Mean_control_ = 22%).

We find no substantial differences between MSM and heterosexual men with regard to caretaking either before (Mean_treated_ = 7%; Mean_control_ = 6%) or after matching (Mean_treated_ = 7%; Mean_control_ = 6%). Likewise, engagement rates across volunteering activities do not differ between the two groups in the unmatched sample (*t* = −0.34) or in the matched sample (*t* = −0.04).

When considering the monetary amounts donated, we find no substantial differences between MSM and heterosexual men before matching (*t* = −1.00). However, after matching we find that MSM donate more money (*t* = 2.17). This corresponds to an approximately 28% ($e^{0.25}-1\approx 28\%$) higher amount among MSM compared to heterosexual men.

The total value of in‐kind donations differs between the two groups before matching (*t* = −2.08). After matching, we no longer find differences between the groups (*t* = 0.14). As a robustness check, we use the nearest neighbour matching approach with replacement. The results remain consistent with the findings reported in Table 2 and can be found in Table A3.

Overall, MSM are more likely to engage in monetary and in‐kind donations than heterosexual men. These differences persist even after applying PS to match MSM with heterosexual men. No differences are observed in caregiving and volunteering rates. After matching, we find no differences between the groups with respect to the amount donated for in‐kind support, suggesting that the differences lie in participation rather than in the intensity or magnitude of contributions.

### RQ 2: Life course dynamics of prosocial engagement among MSM and heterosexual men

Table 3 presents weighted regression (applying inverse probability weights) models estimating the joint effects of sexual orientation and age on prosocial engagement.

**TABLE 3:** Interaction effects of sexual orientation and age on prosocial engagement (inverse probability weights).

	Money donation				In‐kind support				Caretaking		Volunteering	
	(Yes/no)		Amount in € (log)^a^		(Yes/no)		Amount in € (log)^a^		(Yes/no)		(Yes/no)	
	*β*	*p*	*β*	*p*	*β*	*p*	*β*	*p*	*β*	*p*	*β*	*p*
Ref. Heterosexual men												
MSM	0.03	0.947	1.08	0.057	1.31	0.013	0.21	0.666	1.66	0.058	−0.98	0.149
Age	0.03	<0.001	0.22	<0.001	0.01	<0.001	0.03	<0.001	0.02	<0.001	0.01	<0.001
Ref. Heterosexual men × age												
MSM × age	0.001	0.865	−0.02	0.098	−0.02	0.075	−0.003	0.802	−0.02	0.206	0.01	0.256
Intercept	−1.54	<0.001	4.01	<0.001	−1.67	<0.001	4.66	<0.001	−3.97	<0.001	−1.97	<0.001
*N*	9519		4304		9496		1894		9540		9494	
Pseudo‐*R* ^2^	0.03				0.01				0.01		0.01	
*R* ^2^			0.03				0.11					

*Note*: Reported coefficients are based on weighted logistic regression models and log‐coefficients for continuous outcomes (log‐transformed amounts) based on weighted ordinary least squares regressions. Weights are derived using inverse probability weighting. All models include an interaction term between sexual orientation (MSM = 1, heterosexual = 0) and age.

Abbreviation: MSM, men who have sex with men.

^a^ For the models with log‐transformed donation amounts, coefficients can be interpreted as percentage changes in the donation amounts. For binary predictors, the percentage change equals 100 × (e^*β*^ *−* 1) [36].

In the domain of money donation, we find that with increasing age, the likelihood to donate money increases (*β* = 0.03, *p* < 0.001). While there are no differences between MSM and heterosexual men in their likelihood to donate money, we find that MSM donate higher amounts (*β* = 1.08, *p* = 0.057).

For in‐kind support, MSM show a higher likelihood of donating compared to heterosexual men (*β* = 1.31, *p* = 0.013). Age is also positively associated with donation behaviour (*β* = 0.01, *p* < 0.001). Furthermore, the interaction effect (*β* = −0.02, *p* = 0.075) indicates that the difference between MSM and heterosexual men slightly decreases with increasing age. With respect to the log‐transformed amount of in‐kind donations, we find no significant effects of sexual orientation or its interaction with age, while age remains a strong positive predictor. That is, an increase of 1 year in age is associated with approximately 3% increase in the donated amount (*β* = 0.03, *p* < 0.001).

In the domain of caretaking, MSM exhibit higher engagement (*β* = 1.66, *p* = 0.058), and older respondents are more likely to engage in caregiving activities (*β* = 0.02, *p* < 0.001). We find a negative interaction between sexual orientation and age (*β* = −0.02, *p* = 0.206), indicating a decreasing gap between MSM and heterosexual men with increasing age.

For volunteering, we find no differences between the groups or in the interaction of age and sexual orientation. The likelihood of volunteering is, however, increasing with age (*β* = 0.01, *p* < 0.001).

Overall, the findings suggest that MSM are more engaged in several forms of prosocial behaviour than heterosexual men, particularly regarding in‐kind support and caretaking. While we do not find higher engagement rates in money donation, we find that MSM donate more compared to heterosexual men. While age predicts prosocial engagement across models, its moderating effect on the relationship between sexual orientation and prosocial engagement is given only for the amount of donated money and in‐kind support.

## DISCUSSION

Our findings indicate that, after matching, a higher proportion of MSM is engaged in money donations (including amount) and in‐kind support. However, when accounting for interaction effects and applying weights, the differences diminish. These results are consistent with findings from the marketing domain examining the consumption patterns between homosexuals and heterosexuals [7]. The results suggest that MSM appear to be more inclined towards various forms of engagement that involve the contribution of financial or material resources. This may reflect differences in lifestyle structures, opportunity costs or preferred modes of social participation and represents an important distinction for organizations that rely on diverse forms of prosocial involvement.

Drawing on the findings of Studte et al. [8], individuals who engage in one prosocial domain are more likely to be prosocially engaged in other domains. Following this logic, one might expect a high willingness to donate blood among MSM.

The results show that MSM exhibit higher levels of prosociality in domains like monetary and in‐kind donations compared to heterosexual men. This suggests that their categorical exclusion of the past may indeed have excluded a particularly ‘prosocially inclined’ group. In absolute terms, Armstrong et al. [33] highlight that 69% of MSM express interest in donating blood. While many MSM express a donation willingness, common intention–behaviour gaps must be considered. Moreover, translating this prosocial engagement potential of MSM into actual donation rates is limited by structural eligibility constraints. Despite the 2023 reform of the Transfusion Act, MSM in Germany remain disproportionately affected by HIV, accounting for 34.8% of new diagnoses in 2024 compared to 29.5% among all heterosexual transmission categories combined [34]. Consequently, even with individualized risk assessments, a smaller proportion of the MSM population meets the medical criteria for donation. These eligibility rules serve a critical safety function by protecting the blood supply from infections that may not yet be known to potential donors. In addition, MSM are disproportionately affected by sexually transmitted infections, a fact that is also stressed in findings from French plasma collection, which indicate a higher prevalence of transfusion‐transmissible infections in this group [23]. While declining HIV incidence among MSM [34] suggests that eligibility may improve over time, the current epidemiological reality remains a primary barrier that decouples high prosocial motivation from actual donation eligibility.

Our findings offer distinct actionable insights for both blood establishments and broader non‐profit organizations. For blood donation services, the 2023 reform represents an opportunity to stabilize the donor pool but also as an important step towards greater social inclusion for a group that has historically faced exclusion. To increase donor turnout, engagement requires more than a policy change. Literature suggests that blood establishments should move towards neutral advertising to reduce group‐related stigma. Furthermore, to rebuild trust and encourage donation, it is essential to implement diversity sensitivity training for staff to ensure a welcoming and inclusive environment at donation sites [33].

Separately, for non‐profit organizations, MSM represent a relevant target group for monetary donations and in‐kind support. Offering alternative channels of prosocial engagement allows organizations to integrate individuals who are unable to donate blood for medical or regulatory reasons, thereby widening participation in civic and social contribution. From a marketing perspective, targeting young MSM could help foster a sense of loyalty to the organization, encouraging long‐term engagement and commitment. This is important, as previous studies have shown that if individuals are already engaged in prosocial activities, reciprocal engagement is likely [8, 9].

Several limitations must be acknowledged. First, we have analysed MSM engagement in prosocial behaviours as a proxy for blood donation behaviour. Thus, our findings can only be interpreted as the potential for blood donation engagement.

Second, MSM are defined based on self‐reported sexual orientation, which may not be fixed, introducing potential measurement error [24].

Third, the sample shows a substantial imbalance between MSM and heterosexual participants. Three‐hundred and eighty‐eight individuals are classified as MSM and 9454 individuals as heterosexual men. This imbalance reduces statistical power and limits the precision of the estimates for the smaller group, despite applying propensity score matching to improve comparability between groups.

Fourth, the generalizability of our findings may be limited by cultural variations in prosocial motives, which differ significantly between western, educated, industrialized, rich and democratic (‘WEIRD’) contexts like Germany and other global regions [35].

To develop a better understanding of how institutional and regulatory frameworks can facilitate increased participation of MSM in blood donation, future research should assess the impact of eligibility rules, deferral criteria and targeted communications, as they relate to building trust and increasing the willingness to donate among MSM populations. Moreover, it would be worthwhile to investigate whether MSM and heterosexual men differ in the number of concurrent prosocial activities they engage in. The purpose of this research was to identify ways to improve the inclusion and participation of donors in blood donation programmes.

## CONFLICT OF INTEREST STATEMENT

Antonia Leiße is a scholarship holder of the German Red Cross Blood Donation Service North East.

## Data Availability

The data that support the findings of this study are available from the Deutsches Institut für Wirtschaftsforschung (DIW). Restrictions apply to the availability of these data, which were used under license for this study. Data are available from https://www.diw.de/en/diw_01.c.615551.en/research_infrastructure__socio‐economic_panel__soep.html with the permission of DIW.

## APPENDIX 1

**TABLE A1:** Operationalization.

Category	Variable	Definition/description	Operationalization
Comparison groups	Sexual orientation	If someone's sex is male and the sexual orientation is heterosexual	0 = heterosexual men
		If someone's sex is male and the sexual orientation is bi‐ or homosexual	1 = MSM
Outcome	Money donation	Did you donate money last year, in 2019, not counting membership fees?	Dummy variable (1 = yes; 0 = no)
	Amount of money donated	How much money did you donate in total?	Metric (in €)
	In‐kind support	Have you personally provided in‐kind support to relatives or other people outside your household in the last year (2019)?	Dummy variable (1 = yes; 0 = no)
	Amount of in‐kind support donated	What was the total value of in‐kind support?	Metric (in €)
	Volunteering	How many side jobs, including volunteer jobs, do you currently have? Is that a volunteer job?	Dummy variable (1 = yes; 0 = no)
	Taking care	Generated variable based on (a) time spent on care, (b) missed workdays due to care and (c) not working because of care.	Dummy variable (1 = yes; 0 = no)
Socio‐demographic	Age	What is your age?	Metric
	Marital status	Generated: What is your marital status?	Dummy variable (1 = married; 0 = unmarried)
	New federal states	Former east Germany	Dummy variable (1 = married; 0 = unmarried)
	Children	Are there children in your household?	Dummy variable (1 = yes; 0 = no)
	Individual labour earnings last year	Gross annual labour income of individuals aged 16+, including wages, self‐employment, bonuses, and allowances. The variable is used as provided in the dataset (including prior data provider imputation)	Metric (In €)
	Fulltime employment	Do you have a fulltime employment?	Dummy variable (1 = yes; 0 = no)
	Unemployment	Are you unemployed?	Dummy variable (1 = yes; 0 = no)
Psychographic factors	Political interest	How interested are you in politics?	4‐point Likert‐scale (1 = not at all; 4 = very much)
	Risk affinity	How do you see yourself: are you generally a person who is fully prepared to take risks or do you try to avoid taking risks?	11‐point Likert‐scale (0 = not at all willing to take risks; 10 = very willing to take risks)
	Positivity	Positive attitude towards oneself	7‐point Likert‐scale (1 = does not apply at all; 7 = fully applies)
	Reciprocity	Willingness to reciprocate favours	7‐point Likert‐scale (1 = does not apply at all; 7 = fully applies)
	Costly reciprocity	Willingness to incur costs to return help	7‐point Likert‐scale (1 = does not apply at all; 7 = fully applies)
	Perceived social determination	Opportunities in life determined by social circumstances	7‐point Likert‐scale (1 = does not apply at all; 7 = fully applies)
	General worry index	How worried do you feel with respect to the following aspects: general economic development, your own economic situation, your retirement benefits, your health, environmental protection, the impact of climate change, maintaining peace, crime, social cohesion, immigration to Germany, xenophobia, keeping up with technological progress, devaluation of job qualifications, balancing work and life, job security	3‐point Likert‐scale (1= no worries; 2= a few worries; 3= big worries)
	Satisfaction with health	How satisfied are you with your health?	11‐point Likert‐scale (0 = very unsatisfied; 10 = very satisfied)
	Current satisfaction	How satisfied are you currently?	11‐point Likert‐scale (0 = very unsatisfied; 10 = very satisfied)
	Mood index	Please indicate for each feeling how often or rarely did you experience this feeling in the past 4 weeks, namely angry, worried, sad, happy?	5‐point Likert‐scale (1 = very seldom; 5 = very often)
Health‐related factors	Chronic illness	Do you have a chronic illness?	Dummy variable (1 = yes; 0 = no)
	Smoking	Do you smoke?	Dummy variable (1 = yes; 0 = no)
	Sleep	How many hours per day do you sleep during weekdays?	Metric (h/day)
	Body mass index	Generated: Based on weight in kg/(height in m)^2^	Metric

Abbreviation: MSM, men who have sex with men.

## APPENDIX 2 Literature‐based inclusion of covariates

The estimation of the propensity score is informed by research showing that men who have sex with men (MSM) and heterosexual men often differ across socio‐demographic, psychographic and health‐related factors. MSM and heterosexual men differ with respect to socio‐demographics, including age, marital status, income [10, 14], personality traits [37], political interest [38], as well as their health and health‐related behaviour, including substance abuse and depression [39].

**TABLE A2:** Results of propensity score estimation (logit model).

	Money donation		Amount money donation		In‐kind support		Amount in‐kind support		Taking care		Volunteering		
	Sexual orientation (0 = heterosexual men; 1 = MSM)												
	*β*	*p*	*β*	*p*	*β*	*p*	*β*	*p*	*β*	*p*	*β*	*p*
Age	−0.02	<0.000	−0.03	<0.000	−0.02	<0.000	−0.03	<0.000	−0.02	<0.000	−0.02	<0.000
Marital status	−0.80	<0.000	−1.06	<0.000	−0.80	<0.000	−0.81	0.002	−0.80	<0.000	−0.78	<0.000
New federal states	0.45	<0.000	0.48	0.005	0.45	<0.000	0.59	0.005	0.45	<0.000	0.47	<0.000
Children	−1.31	<0.000	−1.69	<0.000	−1.31	<0.000	−1.10	<0.000	−1.31	<0.000	−1.31	<0.000
Individual labour earnings last year	0.00	0.649	0.00	0.447	0.00	0.652	0.00	0.389	0.00	0.653	0.00	0.667
Fulltime employment	−0.18	0.129	−0.24	0.183	−0.19	0.127	−0.09	0.695	−0.18	0.131	−0.18	0.151
Unemployment	−0.24	0.333	0.27	0.525	−0.24	0.330	0.61	0.212	−0.24	0.335	−0.22	0.372
Political interest	0.53	<0.000	0.50	<0.000	0.53	<0.000	0.54	<0.000	0.53	<0.000	0.54	<0.000
Risk affinity	−0.02	0.406	−0.08	0.034	−0.02	0.396	0.03	0.598	−0.02	0.400	−0.02	0.332
Positivity	−0.15	0.002	−0.04	0.599	−0.15	0.002	−0.12	0.217	−0.14	0.003	−0.15	0.002
Reciprocity	0.15	0.045	0.22	0.062	0.15	0.045	0.32	0.038	0.15	0.046	0.16	0.032
Costly reciprocity	0.02	0.710	−0.03	0.631	0.02	0.713	−0.05	0.599	0.02	0.720	0.02	0.692
Perceived social determination	0.04	0.260	−0.03	0.614	0.04	0.252	0.04	0.572	0.04	0.257	0.05	0.221
General worry index	−0.34	0.066	−0.06	0.834	−0.34	0.068	−0.35	0.356	−0.34	0.067	−0.35	0.060
Satisfaction with health	0.03	0.469	0.06	0.227	0.02	0.465	0.03	0.673	0.03	0.455	0.03	0.410
Current satisfaction	0.12	0.006	0.17	0.022	0.12	0.006	0.20	0.023	0.12	0.006	0.12	0.009
Mood index	0.30	0.003	0.49	0.001	0.30	0.003	0.46	0.015	0.30	0.003	0.29	0.004
Chronic illness	0.37	0.003	0.36	0.038	0.37	0.003	0.12	0.584	0.37	0.003	0.37	0.003
Smoking	0.30	0.014	0.40	0.026	0.29	0.014	0.10	0.659	0.30	0.014	0.31	0.009
Sleep	−0.05	0.263	−0.05	0.471	−0.05	0.266	−0.05	0.609	−0.05	0.265	−0.05	0.328
Body mass index	−0.01	0.414	0.00	0.984	−0.01	0.415	0.00	0.990	−0.01	0.420	−0.01	0.486
Intercept	−3.98	<0.000	−4.97	<0.000	−3.99	<0.000	−5.67	0.002	−3.99	<0.000	−4.12	<0.000
*N*	9519		4304		9496		1894		9540		9494	
Pseudo *R* ^2^ before	0.11		0.17		0.12		0.15		0.12		0.12	
Pseudo *R* ^2^ after	0.01		0.01		0.02		0.01		0.02		0.02	
Mean bias before	17.9		21.2		17.9		20.2		17.9		18.1	
Mean bias after	5.1		4.1		5.1		3.7		5.1		5.2	

Abbreviation: MSM, men who have sex with men.

## APPENDIX 3

**TABLE A3:** Comparison of matching algorithms.

Matching algorithm	Effect	SE	*t*‐value	No. of support	Mean bias before	Mean bias after
Money donation						
Kernel	0.09	0.03	3.37	0	17.9	5.1
Nearest neighbour with replacement	0.11	0.04	3.02	0	17.9	3.3
Amount money donation (log)						
Kernel	0.25	0.11	2.17	0	21.2	4.1
Nearest neighbour with replacement	0.35	0.16	2.22	0	21.2	5.2
In‐kind support						
Kernel	0.09	0.02	3.81	0	17.9	5.1
Nearest neighbour with replacement	0.09	0.03	2.68	0	17.9	4.1
Amount in‐kind support (log)						
Kernel	0.02	0.12	0.14	0	20.2	3.7
Nearest neighbour with replacement	0.04	0.19	0.19	0	20.2	8.0
Taking care						
Kernel	0.02	0.01	1.11	0	17.9	5.1
Nearest neighbour with replacement	0.00	0.02	0	0	17.9	3.6
Volunteering						
Kernel	0.00	0.02	−0.04	0	18.1	5.2
Nearest neighbour with replacement	−0.02	0.03	−0.56	0	18.1	4.5

## APPENDIX 4

**TABLE A4:** Group comparison results for engagement in prosocial behaviour before and after matching (nearest neighbour).

		MSM (treated)	Heterosexual men (control)	Diff.	SE	*t*
Money donation (yes/no)	Mean_before_	0.52	0.45	0.06	0.03	2.50*
	Mean_after_	0.52	0.41	0.11	0.04	3.02**
	*N*	381	9138			
In‐kind support (yes/no)	Mean_before_	0.31	0.21	0.11	0.02	5.10***
	Mean_after_	0.31	0.23	0.09	0.03	2.68**
	*N*	381	9115			
Taking care (yes/no)	Mean_before_	0.07	0.06	0.01	0.01	0.88
	Mean_after_	0.07	0.07	0.00	0.02	0.00
	*N*	381	9159			
Volunteering (yes/no)	Mean_before_	0.16	0.17	−0.01	0.02	−0.34
	Mean_after_	0.16	0.18	−0.02	0.03	−0.56
	*N*	377	9117			
Log amount of money donated	Mean_before_	5.12	5.23	−0.11	0.11	−1.00
	Mean_after_	5.12	4.77	0.35	0.16	2.22*
	*N*	198	4106			
Log amount of in‐kind donated	Mean_before_	5.84	6.08	−0.24	0.12	−2.08*
	Mean_after_	5.84	5.80	0.04	0.19	0.19
	*N*	119	1775			

Abbreviation: MSM, men who have sex with men.

* *p* < 0.05;

** *p* < 0.01;

*** *p* < 0.001.

## APPENDIX 5

**TABLE A5:** Quality criteria for matching (applying kernel matching), illustrated using the dependent variable money donation (yes/no).

Matching variable		MSM (treated)	Heterosexual men (control)	%Bias	SPRB	*t*	$p>\left|t\right|$
		Mean	Mean				
Age	Unmatched	45.81	51.87	−36.1	79.7	−6.78	0.000
	Matched	45.81	47.04	−7.3		−0.97	0.334
Marital status	Unmatched	0.32	0.63	−65.9	71.6	−12.41	0.000
	Matched	0.32	0.41	−18.7		−2.56	0.011
New federal states	Unmatched	0.34	0.25	19.9	64.7^a^	3.98	0.000
	Matched	0.34	0.30	7.0		0.94	0.348
Children	Unmatched	0.10	0.32	−55.0	66.9	−9.08	0.000
	Matched	0.10	0.17	−18.4		−2.91	0.004
Individual labour earnings last year	Unmatched	33,179.00	44,698.00	−11.1	68.5^a^	−1.62	0.106
	Matched	33,179.00	36,810.00	−3.5		−0.64	0.521
Fulltime employment	Unmatched	0.48	0.56	−16.2	68.3^a^	−3.10	0.002
	Matched	0.48	0.50	−5.1		−0.70	0.482
Unemployment	Unmatched	0.06	0.05	5.7	74.6^a^	1.16	0.246
	Matched	0.06	0.05	1.5		0.19	0.847
Political interest	Unmatched	2.91	2.61	34.7	71.8	6.73	0.000
	Matched	2.91	2.82	9.8		1.35	0.178
Risk affinity	Unmatched	5.48	5.51	−1.4	92.7^a^	−0.26	0.798
	Matched	5.48	5.49	−0.1		−0.01	0.989
Positivity	Unmatched	5.45	5.73	−21.7	72.2	−4.58	0.000
	Matched	5.45	5.53	−6.1		−0.80	0.427
Reciprocity	Unmatched	6.50	6.39	13.5	70.0^a^	2.47	0.014
	Matched	6.50	6.47	4.0		0.59	0.558
Costly reciprocity	Unmatched	5.61	5.49	9.3	67.4^a^	1.76	0.078
	Matched	5.61	5.57	3.0		0.42	0.674
Perceived social determination	Unmatched	4.56	4.36	13.6	70.3^a^	2.62	0.009
	Matched	4.56	4.50	4.1		0.56	0.578
General worry index	Unmatched	1.82	1.82	−1.6	90.8^a^	−0.31	0.760
	Matched	1.82	1.82	−0.2		−0.02	0.983
Satisfaction with health	Unmatched	6.91	6.83	3.7	92.3^a^	0.73	0.466
	Matched	6.91	6.91	0.3		0.04	0.968
Current satisfaction	Unmatched	7.50	7.60	−6.3	71.5^a^	−1.20	0.231
	Matched	7.50	7.53	−1.8		−0.24	0.807
Mood index	Unmatched	2.38	2.24	20.1	70.8	3.98	0.000
	Matched	2.38	2.34	5.9		0.79	0.431
Chronic illness	Unmatched	0.45	0.39	11.5	63.4^a^	2.22	0.027
	Matched	0.45	0.43	4.2		0.58	0.565
Smoking	Unmatched	0.31	0.25	14.8	74.8^a^	2.93	0.003
	Matched	0.31	0.30	3.7		0.50	0.618
Sleep	Unmatched	6.91	6.92	−1.3	91.5^a^	−0.26	0.792
	Matched	6.91	6.91	−0.1		−0.02	0.988
Body mass index	Unmatched	26.64	27.25	−11.9	76.7^a^	−2.46	0.014
	Matched	26.64	26.78	−2.8		−0.38	0.703

Abbreviation: MSM, men who have sex with men; SPRB, sample percent reduction in bias.

^a^ Interpretation not advised due to <20% pre‐matching bias as standardized bias measures are relative to pre‐matching imbalance and may overstate balance when initial differences are small or uneven [40].

## APPENDIX 6

**TABLE A6:** Quality criteria for matching (applying nearest neighbour matching with replacement), illustrated using the dependent variable money donation (yes/no).

Matching variable		MSM (treated)	Heterosexual men (control)	%Bias	SPRB	*t*	$p>\left|t\right|$
		Mean	Mean				
Age	Unmatched	45.81	51.85	−36.0	98.9	−6.78	0.000
	Matched	45.81	45.88	−0.4		−0.05	0.958
Marital status	Unmatched	0.32	0.63	−65.9	97.5	−12.41	0.000
	Matched	0.32	0.33	−1.7		−0.23	0.816
New federal states	Unmatched	0.34	0.25	19.9	85.4^a^	3.98	0.000
	Matched	0.34	0.32	2.9		0.38	0.700
Children	Unmatched	0.10	0.32	−55.6	95.2	−9.08	0.000
	Matched	0.10	0.09	2.7		0.49	0.625
Individual labour earnings last year	Unmatched	33,179.00	44,698.00	−11.1	50.2^a^	−1.62	0.106
	Matched	33,179.00	38,910.00	−5.5		−1.11	0.267
Fulltime employment	Unmatched	0.48	0.56	−16.2	96.7^a^	−3.10	0.002
	Matched	0.48	0.47	0.5		0.07	0.942
Unemployment	Unmatched	0.06	0.05	5.7	100^a^	1.16	0.246
	Matched	0.06	0.06	0.0		0.00	1.000
Political interest	Unmatched	2.91	2.61	34.7	90.3	6.73	0.000
	Matched	2.91	2.88	3.4		0.47	0.640
Risk affinity	Unmatched	5.48	5.51	−1.4	29.4^a^	−0.26	0.798
	Matched	5.48	5.50	−1.0		−0.13	0.895
Positivity	Unmatched	5.45	5.73	−21.7	77.7	−4.58	0.000
	Matched	5.45	5.39	4.9		0.63	0.529
Reciprocity	Unmatched	6.50	6.39	13.5	50.4^a^	2.47	0.014
	Matched	6.50	6.44	6.7		1.00	0.318
Costly reciprocity	Unmatched	5.61	5.49	9.3	93.4^a^	1.76	0.078
	Matched	5.61	5.60	0.6		0.09	0.931
Perceived social determination	Unmatched	4.56	4.36	13.6	60.3^a^	2.62	0.009
	Matched	4.56	4.48	5.4		0.73	0.464
General worry index	Unmatched	1.82	1.82	−1.6	86.6^a^	−0.31	0.760
	Matched	1.82	1.82	−0.2		−0.03	0.976
Satisfaction with health	Unmatched	6.91	6.83	3.7	−179.5^a^	0.73	0.466
	Matched	6.91	6.69	10.4		1.37	0.172
Current satisfaction	Unmatched	7.50	7.60	−6.3	68.1^a^	−1.20	0.231
	Matched	7.50	7.47	2.0		0.26	0.798
Mood index	Unmatched	2.38	2.24	20.1	54.1	3.98	0.000
	Matched	2.38	2.44	−9.2		−1.22	0.224
Chronic illness	Unmatched	0.45	0.39	11.5	62.9^a^	2.22	0.027
	Matched	0.45	0.47	−4.3		−0.58	0.561
Smoking	Unmatched	0.31	0.25	14.8	84.1^a^	2.93	0.003
	Matched	0.31	0.32	−2.3		−0.31	0.756
Sleep	Unmatched	6.91	6.92	−1.3	−47.4^a^	−0.26	0.792
	Matched	6.91	6.93	−2.0		−0.25	0.801
Body mass index	Unmatched	26.64	27.25	−11.9	69.7^a^	−2.46	0.014
	Matched	26.64	26.45	3.6		0.51	0.613

Abbreviations: MSM, men who have sex with men; SPRB, sample percent reduction in bias.

^a^ Interpretation not advised due to <20% pre‐matching bias as standardized bias measures are relative to pre‐matching imbalance and may overstate balance when initial differences are small or uneven [40].
